# Status of insecticide resistance in high-risk malaria provinces in Afghanistan

**DOI:** 10.1186/s12936-016-1149-1

**Published:** 2016-02-18

**Authors:** Mushtaq Ahmad, Cyril Buhler, Patricia Pignatelli, Hilary Ranson, Sami Mohammad Nahzat, Mohammad Naseem, Muhammad Farooq Sabawoon, Abdul Majeed Siddiqi, Martijn Vink

**Affiliations:** HealthNet TPO, Kabul, Afghanistan; ORDiagnostics, 10 rue Irénée Blanc, 75020 Paris, France; Liverpool School of Tropical Medicine, Pembroke Place, Liverpool, L3 5QA UK; National Malaria and Leishmaniasis Control Programme, Ministry of Public Health, Kabul, Afghanistan; HealthNet TPO, Lizzy Ansinghstraat 163, 1072 RG Amsterdam, The Netherlands

**Keywords:** Insecticide resistance, Afghanistan, *Anopheles stephensi*, *Knockdown resistance* (kdr) mutation, Organochlorides, Pyrethroids, Carbamates, Organophosphates

## Abstract

**Background:**

Insecticide resistance seriously threatens the efficacy of vector control interventions in malaria endemic countries. In Afghanistan, the status of insecticide resistance is largely unknown while distribution of long-lasting insecticidal nets has intensified in recent years. The main objective of this study was thus to measure the level of resistance to four classes of insecticides in provinces with medium to high risk of malaria transmission.

**Methods:**

Adult female mosquitoes were reared from larvae successively collected in the provinces of Nangarhar, Kunar, Badakhshan, Ghazni and Laghman from August to October 2014. WHO insecticide susceptibility tests were performed with DDT (4 %), malathion (5 %), bendiocarb (0.1 %), permethrin (0.75 %) and deltamethrin (0.05 %). In addition, the presence of *kdr* mutations was investigated in deltamethrin resistant and susceptible *Anopheles stephensi* mosquitoes collected in the eastern provinces of Nangarhar and Kunar.

**Results:**

Analyses of mortality rates revealed emerging resistance against all four classes of insecticides in the provinces located east and south of the Hindu Kush mountain range. Resistance is observed in both *An. stephensi* and *Anopheles culicifacies*, the two dominant malaria vectors in these provinces. *Anopheles superpictus* in the northern province of Badakhshan shows a different pattern of susceptibility with suspected resistance observed only for deltamethrin and bendiocarb. Genotype analysis of *knock down resistance* (*kdr*) mutations at the voltage-gated channel gene from *An. stephensi* mosquitoes shows the presence of the known resistant alleles L1014S and L1014F. However, a significant fraction of deltamethrin-resistant mosquitoes were homozygous for the 1014L wild type allele indicating that other mechanisms must be considered to account for the observed pyrethroid resistance.

**Conclusions:**

This study confirms the importance of monitoring insecticide resistance for the development of an integrated vector management in Afghanistan. The validation of the *kdr* genotyping PCR assay applied to *An. stephensi* collected in Afghanistan paves the way for further studies into the mechanisms of insecticide resistance of malaria vectors in this region.

**Electronic supplementary material:**

The online version of this article (doi:10.1186/s12936-016-1149-1) contains supplementary material, which is available to authorized users.

## Background

Malaria is a significant health problem in Afghanistan with more than eight million people still living in high transmission areas [1]. Malaria transmission is seasonal with the vast majority of cases recorded from June to November [2]. The Hindu Kush mountain range and the arid climate in the south result in transmission areas restricted to snow-fed river valleys and irrigated zones below 2000 m above sea level [3]. *Plasmodium vivax* accounts for 95 % and *Plasmodium falciparum* for 5 % of the malaria cases. In 2013, 39,263 confirmed malaria cases were recorded [1] and, in endemic areas, the prevalence of *P. vivax* is above 5 % [3].

Amongst the numerous Anopheles species present in the country, the principal malaria vectors are *Anopheles superpictus*, *Anopheles culicifacies*, *Anopheles hyrcanus*, *Anopheles pulcherrimus* and *Anopheles stephensi* [4]. The extensive DDT-based spraying programmes conducted from 1950s to early 1970s resulted in a near eradication of *An. superpictus,* the main malaria vector in the country. Unfortunately, *An. stephensi* and to a lesser extent *An. culicifacies* had become resistant to DDT in the south and eastern provinces bordering Pakistan and have replaced the *An. superpictus populations* in these regions [2, 4]. The development of new cultivated areas in the North also led to the selection or re-emergence of the outdoor resting *An. pulcherrimus* and *An. hyrcanus* populations which represent now the two main malaria vectors observed in the rice fields of Kunduz province [3, 5] and in the wider region including the southern part of Tajikistan [6]. Due to its ability to survive at relatively high altitude, *An. superpictus* seems to be now mostly restricted to freshwater breeding sites in valleys of the Hindu Kush mountain range [7].

Vector control interventions are cost effective and essential measures to control malaria [8, 9]. The lack of an effective malaria vaccine and the presence or emergence of resistance to existing anti-malarial drugs further increases reliance on indoor residual spraying (IRS) and distribution of long-lasting insecticidal nets (LLINs) to control malaria vectors. Between 1949 and 1973 IRS campaigns have been conducted across the country, first with DDT and then (when this pesticide lost is effectiveness) with malathion. In the years thereafter, small-scale spraying campaigns were conducted with insecticides supplied by the USSR, Iraq and the UK, but after the Soviet invasion in 1979 IRS campaigns in the country stopped altogether [4]. Since 2001 IRS has been implemented occasionally but only on a local scale to control malaria epidemics. In the beginning of the 1990s insecticide-treated nets (ITNs) were introduced in Afghan refugee camps in Pakistan and from 1992 in Afghanistan itself. The ITNs were treated—and later retreated—with deltamethrin, permethrin or alpha-cypermethrin. From 2007, ITNs were replaced by LLINs and a universal free coverage of LLINs was implemented through house-to-house distribution campaigns. Between 2007 and 2015, more than nine million deltamethrin-treated LLINs were distributed to households in the main malaria-endemic provinces across the country as defined by a risk stratification map developed by the WHO and the Ministry of Public Health (MoPH) [10]. Further LLINs distribution is still ongoing in the country and is coordinated by a Vector Borne Disease Task Force at the Ministry of Public Health.

Only four classes of insecticides are currently approved for IRS: organochlorides, organophosphates, carbamates and pyrethroids [11]. The situation with LLINs is even more problematic as pyrethroids are the only insecticide class approved for safety reasons and efficacy [12]. The repeated use of the same insecticides combined with agriculture pesticide usage has maintained a selection pressure amongst mosquito populations leading inevitably to the development of insecticide resistance in many African malaria endemic countries [13]. Resistance to several insecticides have also been reported in the Middle East region including DDT resistance in Iran [14] and pyrethroid resistance in *An. stephensi* in Dubai [15], although such monitoring is less developed than in the sub-Saharan or East African region (see the current status of insecticide resistance worldwide on the IR mapper interactive tool [16]). Data on insecticide susceptibility in Afghanistan is still very limited and further complicated by the number of endemic Anopheles species present in the country. The latest and only data available so far come from a susceptibility study conducted by the National Malaria and Leishmaniasis Control Programme (NMLCP) of the MoPH in 2010 that showed a reduction in susceptibility to pyrethroids, carbamates and organochlorines especially in the eastern province of Nangarhar [10]. Accurate measures of insecticide resistance in Afghanistan are thus essential to aid the Vector Borne Disease Task Force with an evidence base to evaluate current vector management interventions, raise awareness in case of increased resistance to specific insecticides and adapt local strategies based on mosquito population dynamics.

**Fig. 1:**
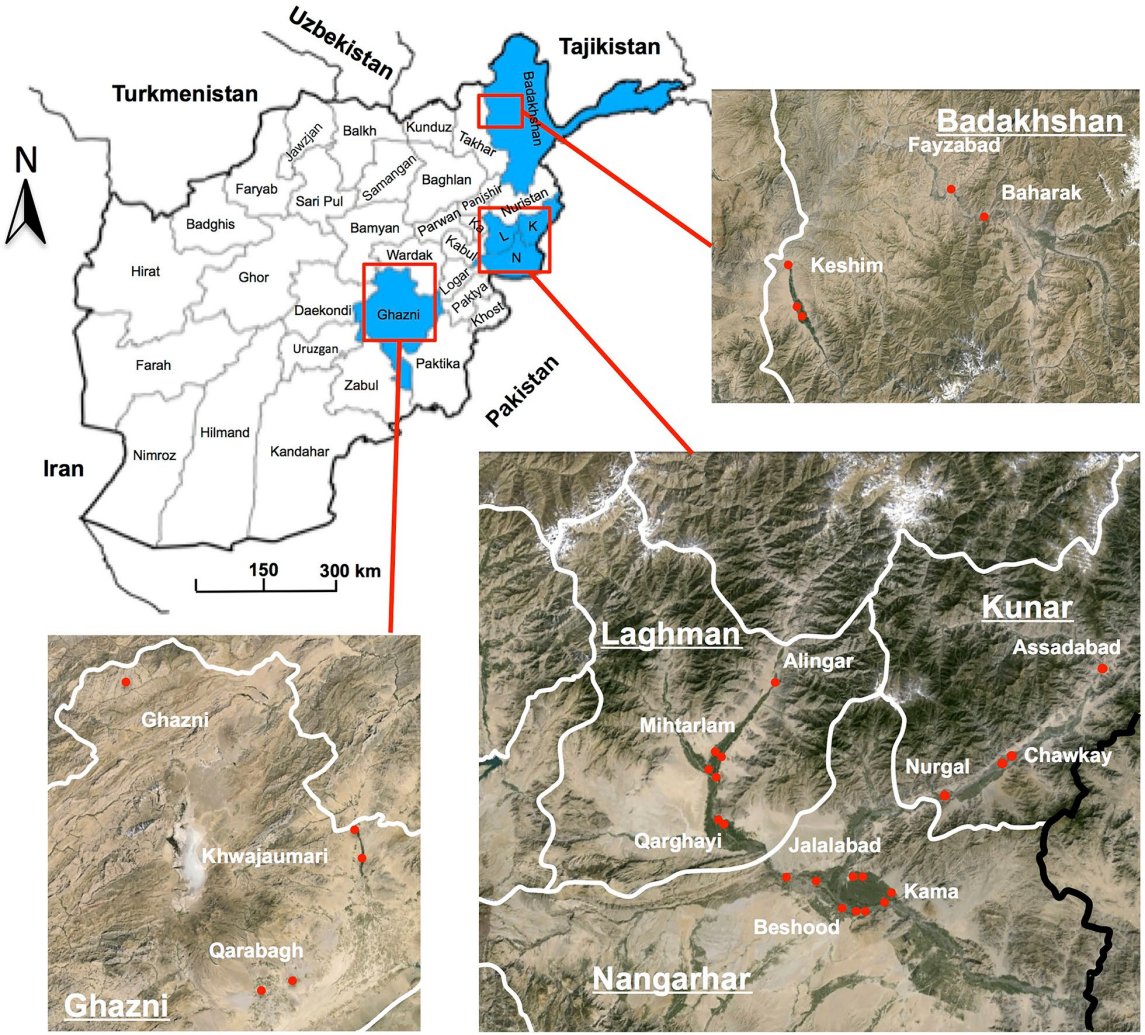
Map showing the study sites in Badakhshan, Laghman, Nangarhar, Ghazni and Kunar

With the growing threat and challenges posed by insecticide resistance in malaria endemic countries, the WHO and Roll Back Malaria have developed the global plan for insecticide resistance management (GPIRM) [13]. Thus, in agreement with the recommendations of the GPIRM, a study was developed to survey the level of resistance in a selection of Afghan provinces. A recent malaria risk stratification at the district level was used to select districts in five high risk malaria provinces for this study: the eastern provinces of Laghman, Nangarhar and Kunar known for the highest rate of *P. falciparum* malaria transmission, the southern province of Ghazni and the northern province of Badakhshan (Fig. 1). As recommended by the WHO, a separate study was implemented to gather information on the underlying mechanisms of resistance. This study focused on target site resistance by assessing the presence of *knock down resistance* (*kdr*) mutations in the voltage-gated channel gene using an allele specific PCR approach previously developed for *An. stephensi* in India [17].

## Methods

### Study sites

Larvae collections were conducted successively in the eastern provinces of Nangarhar, Kunar and Laghman, the northern province of Badakhshan and the southern province of Ghazni from August to October 2014. In order to obtain a good representation of insecticide susceptibility at the provincial level at least three districts described as medium to high-risk malaria transmission by the NMLCP were selected within each province [10]. Locations of the study sites are indicated in Fig. 1 and ecological characteristics of each collection site are provided in Table 1.

**Table 1:** Habitat description and localization of sample collection sites

Province	District	Malaria risk strata	Village	Habitat type	Elevation (m)	Latitude	Longitude
Nangarhar	Behsood	1	Banaghar	Rice field	551	34°26′25.83″N	70°28′56.11″E
	Behsood	1	Samar Khel	Swamps River	525	34°22′39.58″N	70°34′50.33″E
	Behsood	1	Saracha	River/streams	540	34°23′13.70″N	70°32′23.52″E
	Jalalabad	1	Bagrami	Pond/river stream	571	34°26′49.08″N	70°24′24.94″E
	Kama	1	Banajur	Rice field	545	34°26′55.65″N	70°35′6.17″E
	Kama	1	Sabir Kalay	Swamps	517	34°24′40.64″N	70°38′23.19″E
	Kama	1	Sangar Sary	Swamps	510	34°24′8.86″N	70°38′30.71″E
Laghman	Alingar	1	Kanda Rajaee	River stream	907	34°49′32.44″N	70°21′40.48″E
	Alingar	1	Nowra	River stream	738	34°40′29.38″N	70°14′14.98″E
	Mihtarlam	1	Badee Abad	River stream	735	34°40′15.36″N	70°14′3.79″E
	Mihtarlam	1	Mihtarlam	Pond	757	34°40′24.27″N	70°12′59.50″E
	Mihtarlam	1	Qala-E-Jogi	River stream	709	34°37′59.12″N	70°13′51.92″E
	Mihtarlam	1	Qarghae	River stream	644	34°32′53.46″N	70°14′29.18″E
	Mihtarlam	1	Tirgari	River stream	735	34°38′41.03″N	70°12′36.20″E
	Qarghayi	1	Lal Khana Abad	Pond	644	34°32′44.87″N	70°14′29.31″E
	Qarghayi	1	Swati	River stream	635	34°32′32.76″N	70°15′13.73″E
	Qarghayi	1	Tarrang	River stream	641	34°32′40.76″N	70°14′41.06″E
Kunar	Asadabad	1	Asadabad	Pond	830	34°52′51.89″N	71°9′37.38″E
	Asadabad	1	Asadabad	Pond	830	34°52′51.89″N	71°9′37.38″E
	Nurgal	1	Nurgal	River stream	658	34°36′45.70″N	70°46′31.76″E
	Chawkay	1	Babur	Pond/river stream	711	34°41′26.04″N	70°56′7.88″E
Ghazni	Ghazni	2	Koshkak	Pond	2962	34°5′45.61″N	67°30′42.61″E
	Ghazni	2	Pasar	Pond	2214	33°36′32.81″N	68°25′17.71″E
	Qarabagh	2	Mushaki	Water puddle	2056	33°13′49.36″N	68°11′27.53″E
	Qarabagh	2	Pol-E-Qalacha	River stream	2106	33°11′25.23″N	68°4′47.88″E
	Khwajaumari	2	Deh Daran	River stream	2295	33°41′52.34″N	68°23′20.05″E
Badakhshan	Baharak	2	Baharak	River stream	1284	37°3′6.96″N	70°40′15.95″E
	Baharak	2	Baharak village	Pond	1300	37°3′8.43″N	70°41′24.42″E
	Fayzabad	1	Fayzabad	River stream	1190	37°7′0.96″N	70°34′30.99″E
	Fayzabad	1	Qaria Reggy	Water puddle	1218	37°7′14.02″N	70°34′26.30″E
	Keshim	1	Gundum Qul	River stream	1014	36°47′17.86″N	70°6′49.25″E
	Keshim	1	Jarr-E-Haji Baba	Water puddle	987	36°48′34.04″N	70°5′51.93″E

### Larval collection and mosquito rearing

In each province, immature stage mosquitoes (larvae or pupae) were collected from breeding sites located within a 2 to 3-km radius in ecological habitats where the probability to find larvae was high (river stream, rice fields, water puddles or other standing water areas). Sites with the highest densities were used for sampling to obtain enough test subjects for the susceptibility assays.

Larvae samples collected in Nangarhar, Laghman and Kunar were raised to the adult stage in an insectary located in Jalalabad. To avoid high mortality rate of larvae during transportation, makeshift insectaries were established in dedicated rooms at district hospitals in the provinces of Badakhshan and Ghazni. In all laboratory settings, temperatures were kept at 25 ± 2 °C and relative humidity at 75 ± 10 %. Larvae were reared in enamel trays containing water with yeast powder and powdered fish food supplements. Following pupation, samples were placed in a small bowl with water and transferred to closed cages for their emergence into adults.

### Mosquito identification

Anopheles mosquitoes were identified morphologically at the adult stage using Glick’s identification keys [18].

### Insecticide susceptibility assays

Insecticide susceptibility tests were carried out using the WHO bioassay [17]. The following diagnostic concentrations of insecticides were used: 4 % DDT, 5 % malathion, 0.1 % bendiocarb, 0.75 % permethrin and 0.05 % deltamethrin. Oil-impregnated papers were used as controls. Test kits and insecticide control oil-impregnated papers were purchased from the Universiti Sains Malaysia (Penang, Malaysia). Filter papers integrity was confirmed using a laboratory-reared *An. stephensi* strain susceptible to the four classes of insecticides. Susceptibility tests were performed using 3–4 days old female mosquitoes. At least 100 test mosquitoes (20–25 mosquitoes per replicates) and 50 female control mosquitoes (2 replicates) were exposed for 1 h to each of the insecticide impregnated papers and were then transferred to recovery tubes with a 10 % glucose cotton-impregnated solution. Mortality was recorded 24 h post exposure. Average mortality was calculated for each insecticide and corrected using Abbot’s formula [19] if the observed mortalities in the control tests were between 5 and 20 %. Tests were discarded if mortality in the control tube was above 20 %. WHO criteria were used to assess susceptibility to each insecticide [13]. A mortality rate below 90 % was indicative of resistance while mortality above 98 % indicates susceptibility. Mortality between 90 and 97 % was suggestive of resistance in the population. In total, 224 susceptibility assays including 58 control assays were performed during this study.

For DDT, deltamethrin and permethrin, knock down rate was recorded at 10, 15, 20, 30, 40, 50 and 60 min in the presence of the corresponding insecticide. After 60 min mosquitoes were transferred to the recovery tube and a last count of the number of knocked down mosquitoes was made at 80 min. A mosquito was considered knocked down if it was unable to stand or fly in a coordinated way.

### Knock down resistance allele genotyping

DNA was extracted from 137 individual mosquitoes following WHO bioassays against deltamethrin (15 alive and 50 dead mosquitoes from Nangarhar and 21 alive and 51 dead mosquitoes from Kunar) using the Qiagen DNeasy blood and tissue kit. *Kdr* genotyping in the Domain II S6 segment of voltage-gated channel gene was performed by two allele-specific PCRs according to the method developed by Singh et al. [17]. The first PCR discriminates the allele 1014F from wild type or 1014S, and the second PCR discriminates 1014S from wild type allele.

### Data and statistical analysis

Cumulative curves of mortality and KDT_50_ and KDT_90_ were calculated with a log time-probit model using Qcal [20]. 2 × 2 contingency tables were used to test for association between presence of the *kdr* allele and survival to deltamethrin in bioassays.

## Results

### Larval identification and habitat documentation

With the exception of Kama district in Nangarhar, the breeding sites visited were mainly uncultivated area corresponding to river banks, ponds or standing water (see summary of ecological habitats in Table 1). All samplings were conducted at altitudes below 2000 m above sea level (asl) with the exception of Ghazni district where *An. stephensi* and *An. superpictus* larvae were collected at altitudes up to 2900 m asl.

**Fig. 2:**
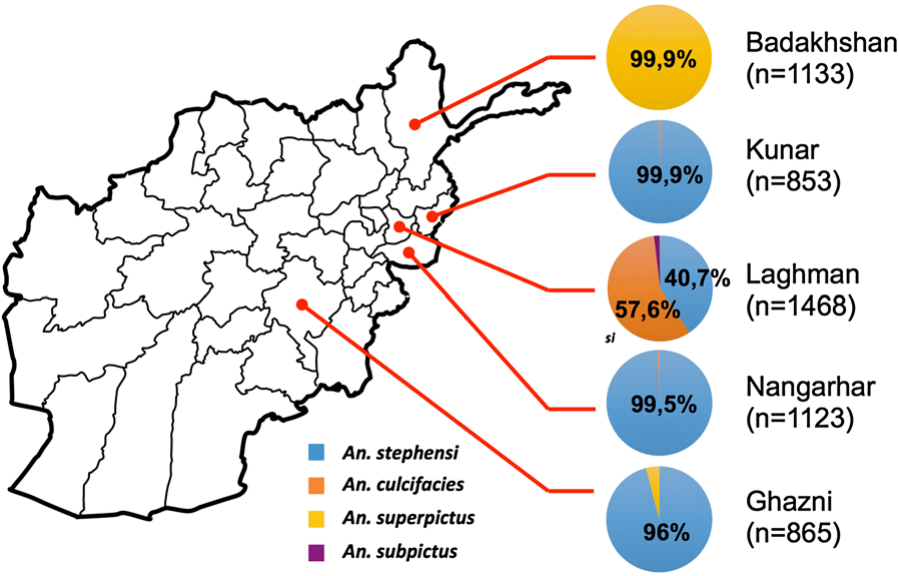
Distribution of the vector species in the selected provinces

In total, 8834 larvae were collected in the five provinces including 2880 larvae belonging to the Culex group (see Additional file 1). Amongst Anopheles species, *An. stephensi* was the dominant species (61.9 %) followed by *An. culicifacies* (20.9 %) and *An. superpictus* (16.3 %). Other marginal species found during this study were *An. splendidus*, *An. nigerimus* and *An. subpictus* (all below 1 %). The distribution of Anopheles species in each of the provinces is presented in Fig. 2. *An. stephensi* was isolated in the provinces south of the Hindu Kush mountain range: in the eastern province of Nangarhar, Kunar and Laghman and in the southern province of Ghazni, as previously documented [4]. Larvae collection in Laghman showed a mixed composition with coexistence of *An. culicifacies* along with *An. stephensi*. *An. superpictus* was isolated in Badakhshan consistent with other observations of its presence in the southern parts of Tajikistan bordering Afghanistan [6]. Overall the malaria vector species identified in this study are consistent with previous bionomic observations performed in Afghanistan [10].

### Insecticide susceptibility

**Fig. 3:**
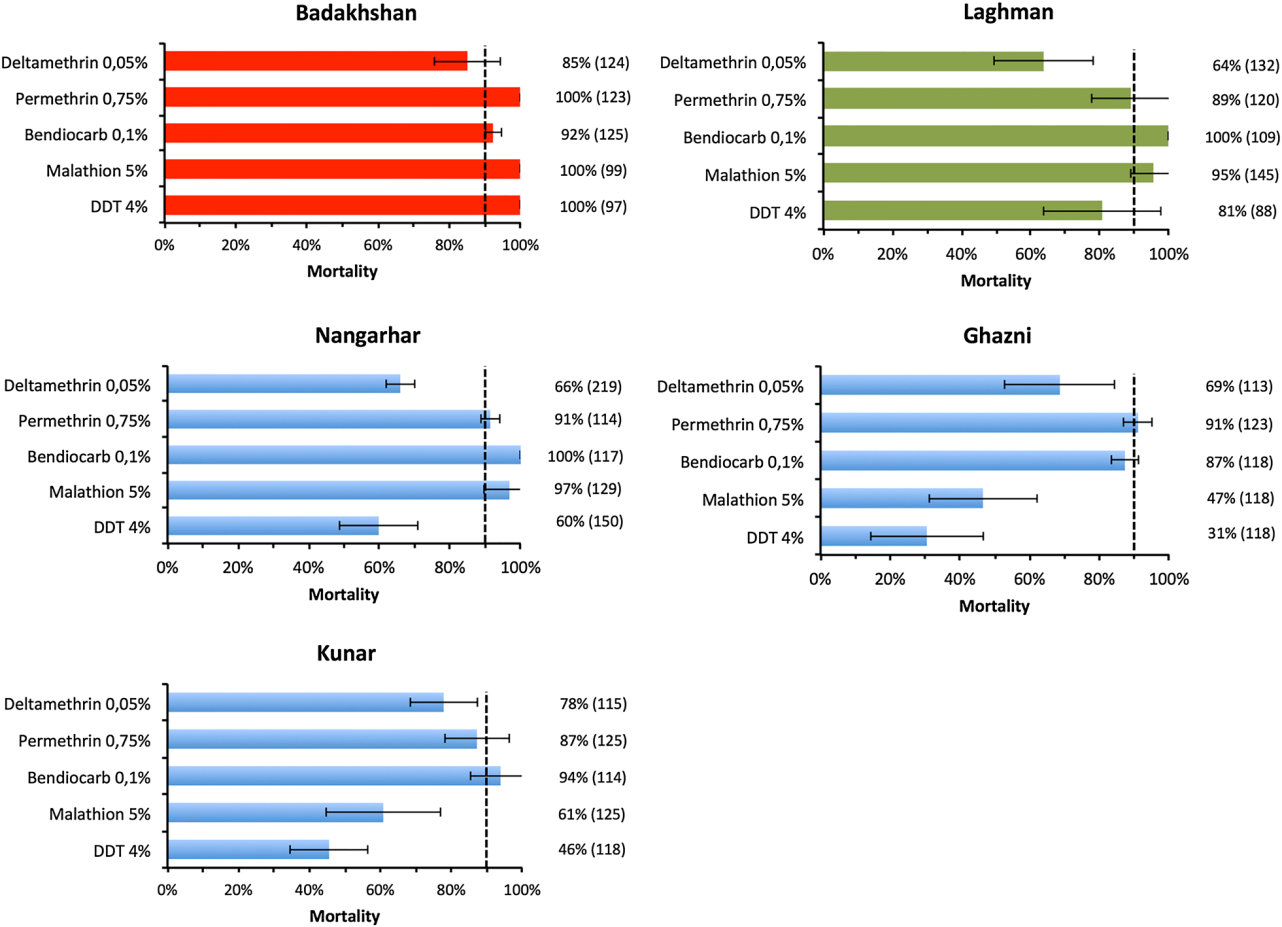
Percentage mortality (±SD) in the five selected provinces. The *dashed lines* correspond to the limit for resistance as defined by WHO criteria [21]. Dominant species tested were *An. stephensi* in Kunar, Ghazni and Nangarhar (shown in *blue*), *An. superpictus* in Badakhshan (*red*) and *An. culicifacies* in Laghman (*green*). The number of mosquitoes used for each bioassay is indicated on the *right*

A total of 2049 female mosquitoes, reared from larvae collected in each province, were exposed to insecticides belonging to the four WHO approved classes. Average mortality rates for the dominant species are presented in Fig. 3. Resistance to deltamethrin was observed for *An. stephensi* (in Nangarhar, Kunar and Ghazni) and *An. culicifacies* (in Laghman) using a threshold of 90 % mortality for resistance confirmation as set by WHO criteria [21]. *Anopheles superpictus* in the northern province of Badakhshan showed also incipient pyrethroid resistance with deltamethrin. Resistance to permethrin is less evident as average mortality rates are near or above 90 % for *An. stephensi* and *An. culicifacies,* whereas *An. superpictus* in Badakhshan remains susceptible.

DDT resistance was observed for *An. stephensi* (in Nangarhar, Kunar and Ghazni) and *An. culicifacies* in Laghman. However, in Badakhshan *An. superpictus* remains susceptible to DDT. The three dominant mosquito species analysed in this study remain largely susceptible to the carbamate insecticide bendiocarb. Finally, contrasting susceptibilities among malaria vectors were observed for malathion as *An. superpictus* and *An. culicifacies* were susceptible whereas resistance was detected for *An. stephensi* mosquitoes collected in Ghazni and Kunar.

**Fig. 4:**
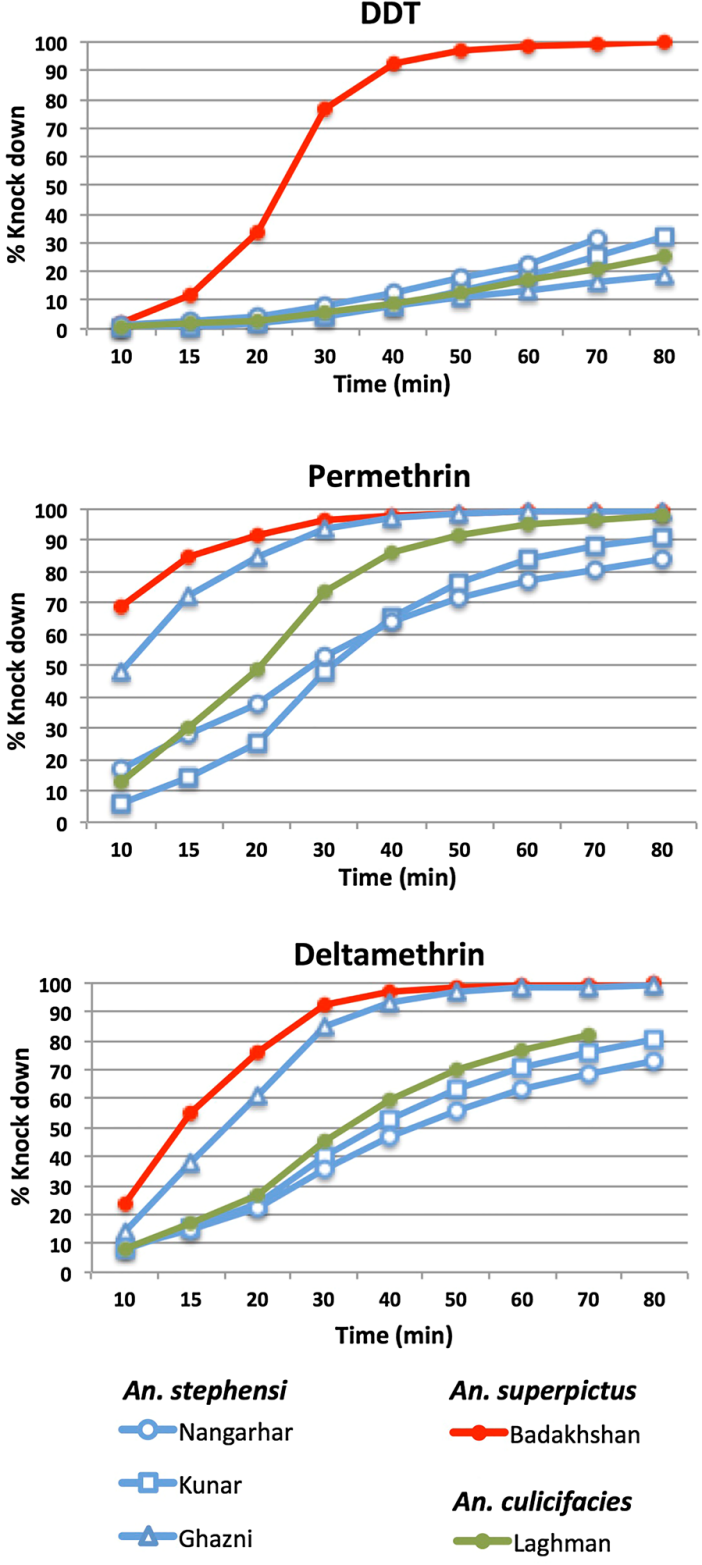
Cumulative knock down rates for DDT (4 %), permethrin (0.75 %) and deltamethrin (0.05 %)

The difference in DDT susceptibility between *An. superpictus* mosquitoes and other malaria vectors was further confirmed by knock down rate analysis (see Fig. 4; Additional file 2). Whereas 90 % of *An. superpictus* are knocked down after less than 40 min in the presence of DDT in Badakhshan (KDT_90_ = 37.5 min, CI 95 % 3.548–3.702), more than 50 % of *An. stephensi* or *An. culicifacies* mosquitoes seems unaffected by this insecticide after 80 min, with KDT_50_ ranging from 100 to 230 min.

Differences in knock down rates against pyrethroids were also observed between *An. stephensi* collected in Ghazni and the same species collected in the eastern provinces of Nangarhar and Kunar despite similar 24 h mortality rates. For example, knock down rates in the presence of deltamethrin is two to three times faster is Ghazni than in Nangarhar or Kunar (KDT_50_ = 17.5 min, CI 95 % 2.787–2.931; KDT_50_ = 43.1 min, CI 95 % 3.660–3.865 and KDT_50_ = 37.3 min, 3.534–3.707, respectively). These variations could suggest different mechanisms of resistance involved in the emerging susceptibility of *An. stephensi* to pyrethroids in these provinces.

### *kdr* genotyping

**Fig. 5:**
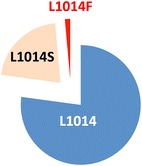
Combined allelic frequencies of L1014 (wild type), 1014F and 1014S alleles from larvae collected in Nangarhar and Kunar provinces

128 of the 137 mosquitoes were successfully genotyped for the *kdr* alleles indicating that the method developed by Singh et al. [17] in India can also be performed on *An. stephensi* mosquitoes collected in Afghanistan. In both sites studied, Kunar and Nangarhar, the wild type 1014L allele was the most prevalent allele followed by 1014S and 1014F *kdr* mutations (Fig. 5). No *kdr* homozygotes were detected, the serine and phenylalanine allele were found as heterozygotes with the wild type. When data from both sites are combined there is a significant association between the presence of either *kdr* allele and phenotype (p < 0.05) although the odds ratios are low (2.24). The finding that only 44 % (15/34) of the bioassay survivors possessed a *kdr* mutation suggests that other resistance mechanisms are also present in these populations.

## Discussion

Gathering bionomic information on endemic malaria vectors is an essential component for the development of an effective vector management plan. Previous entomological studies performed in Afghanistan have highlighted the diversity of Anopheles species present in the country [4, 5]. This study confirmed that *An. stephensi* and to a lesser extent *An. culicifacies* are the dominant species in the provinces located in the east (Nangarhar, Laghman and Kunar) and south (Ghazni) of the Hindu Kush mountain range. *An. superpictus* was the only species identified in the northern province of Badakhshan. *Anopheles hyrcanus* and *An. pulcherrimus* have previously been identified in rice fields in the northern province of Kunduz in 2005–2006 [5], but were not detected in the current study. This uneven representation of Anopheles species in northeastern provinces may reflect differences in the densities of irrigated and cultivated areas, in addition to preferences for specific types of ecological habitat within each province [22].

It has been well documented that malaria in Afghanistan is endemic to areas that are below 2000 m asl although episodes of *P. falciparum* malaria may occur in areas above 2400 m asl [23]. The presence of *An. stephensi* and *An. superpictus* at high altitudes (up to 2900 m asl) in Ghazni is therefore not surprising and highlights the distribution of the vectors to a variety of environmental conditions. With the exception of rice fields in Nangarhar, the three dominant species identified in this study (*An. superpictus*, *An. culicifacies* and *An. stephensi*) were collected from freshwater breeding site and ponds. As larvae collections were performed during 2–3 weeks successively in each province, the relative representation of Anopheles species in each of the provinces may well vary during the malaria transmission season.

Adults reared from the dominant larvae species found in each of the five provinces (*An. stephensi* in Nangarhar, Kunar and Ghazni, *An. culicifacies* in Laghman and *An. superpictus* in Badakhshan) were used as test subjects to measure the level of insecticide resistance using the WHO test procedure [21]. Overall, insecticide resistance was observed (or highly suspected) to at least pyrethroids and DDT. The situation in Badakhshan is different from the other provinces as *An. superpictus* mosquitoes from that province were susceptible to all insecticides tested with the exception of deltamethrin for which emerging resistance is suspected.

It is now evident that pesticide usage from agriculture activities and increased coverage of LLINs in vector control can directly select for insecticide resistance [24, 25]. Despite a lack of data on precise pesticide usage in Afghanistan, it is likely that pest control activities have consequences on mosquito populations and could potentially lead to cross-resistance with the insecticides used in malaria vector control activities. Such selection pressure could be even more exacerbated in Afghanistan where potential mosquito breeding sites and rice fields are closely associated and restricted to valleys. A limited number of irrigation infrastructures and less agricultural areas compared to other provinces could thus explain the relative susceptibility observed for *An. superpictus* mosquitoes collected in Badakhshan, although it is possible that this species is more sensitive to the standard doses of insecticide used in this study. Additive or synergistic effects with pesticides in provinces with more intensive agriculture and irrigation could also be determinant in the observed resistance, as cross-resistance has previously been described in other countries [24]. Beside the dispersion of resistant mosquitoes from neighbouring provinces, the observed resistance in Ghazni could be an example of such cross-resistance as bed nets distribution has been implemented only recently (HealthNet TPO, personal data).

Massive distribution of deltamethrin-impregnated LLINs in Afghanistan over the past decade is a likely contributor to the emerging deltamethrin insecticide resistance that was observed. The LLINs that were distributed since 2007 are impregnated with deltamethrin (PermaNet 2.0). In Badakhshan, irrigation is less developed compared to other Afghan provinces and agriculture is more oriented towards pastoral activities. The resistance to pyrethroid (at least deltamethrin) observed for *An. superpictus* Badakhshan is thus most likely a direct consequence of the bed net distribution campaigns as crop production and irrigation infrastructure is less developed in this province.

Understanding the mechanisms of resistance is essential to adapt vector control strategies and helps predict the origin (new emergence versus migration of resitant populations) and likely impact of resistance [13]. So far analysis of the underlying mechanisms of resistance has not been done in Afghanistan. Although a method has been developed to genotype *kdr* mutations in *An. stephensi* mosquitoes in India [17], DNA sequence variations at the *vgc* locus may have reduced the fidelity of this genotyping protocol. The implemented study aimed initially to test if this method can be effectively applied using *An. stephensi* mosquitoes collected in Afghanistan. PCR amplicons were successfully obtained at the kdr locus in 93 % of the mosquitoes tested (128 out of 137) indicating that this methodology can be used with no additional optimization of the reaction conditions. Therefore, this is a new tool available for vector and malaria control programmes in Afghanistan to understand and follow up acquired resistance against pyrethroids.

In addition, this study provided information on the relative distribution of *kdr* mutations relative to the wild type allele. The pattern of L1014S and L1014F mutations is similar to observations in India with L1014S being more prevalent than L1014F. No homozygote *kdr* mutations were observed, although a relatively low sample size (restricted to the eastern provinces of Nangarhar and Kunar) was used in this study. Finally, as some deltamethrin-resistant mosquitoes do not express mutated forms of the *vgc* gene, other mechanisms of resistance must be considered to explain this phenotype.

## Conclusions

This study showed that insecticide resistance is now emerging within malaria vectors in Afghanistan and highlights the importance of establishing an insecticide resistance management plan [26]. The observation that the pattern of insecticide susceptibility varies amongst the different Anopheles species and ecological contexts advocates for additional bionomic studies associated with insecticide resistance monitoring in all malaria endemic provinces. The impact of the current levels of resistance on the efficacy of LLINs is not known. However, as theory and practice both indicate that levels of pyrethroid resistance in malaria vectors will continue to increase, this must be carefully monitored and complementary interventions implemented if there is indication that the protective efficacy of LLINs is diminished by insecticide resistance in Afghanistan.

## Additional files

10.1186/s12936-016-1149-1 Species distribution in each district and provinces.
10.1186/s12936-016-1149-1 50 % and 90 % knock down time in minutes (KDT50 and KDT90 respectively) for DDT, permethrin and deltamethrin.

## Abbreviations

asl: above sea level
DDT: dichlorodiphenyltrichloroethane
GPIRM: global plan for insecticide resistance management
IRS: indoor residual spraying
kdr: knock down resistance
KDT_50_: 50 % knock down time
KDT_90_: 90 % knock down time
LLIN: long-lasting insecticidal net
MoPH: Ministry of Public Health
NMLCP: National Malaria and Leishmaniasis Control Programme
PCR: polymerase chain reaction
WHO: World Health Organization
