# Supplementary material for: Status of insecticide resistance in high-risk malaria provinces in Afghanistan
Source: Malar J. 2016 Feb 18;15:98. doi: 10.1186/s12936-016-1149-1 (PMC4758152; doi:10.1186/s12936-016-1149-1)
Supplement: Supplementary file 1 — 10.1186/s12936-016-1149-1 Species distribution in each district and provinces. [file 12936_2016_1149_MOESM1_ESM.docx]

| Table S1: Species distribution in each and district and provinces | | | | | | | | | | |
| --- | --- | --- | --- | --- | --- | --- | --- | --- | --- | --- |
|  |  | *Anopheles* | | | | | | *Culcidae* | |  |
| Province | **District** | *A. stephensi* | *A. culicifacies* | *A. splindidus* | *A. nigerimus* | *A. superpictus* | *A. subpictus* | | *Culex* |  |
| Nangarhar | Behsood | 98% (427) | 2% (5) |  |  |  |  | | 129 |  |
|  | Jalalabad | 100% (367) |  |  |  |  |  | | 94 |  |
|  | Kama | 100% (513) |  |  |  |  |  | | 228 |  |
| Laghman | Mihtarlam | 33.5% (225) | 65.8% (442) |  |  |  | 0.7% (4) | | 251 |  |
|  | Alingar | 41.0% (388) | 58.1% (550) |  |  |  | 0.9% (8) | | 301 |  |
|  | Qarghayi | 44.6% (322) | 48.5% (350) | 0.1% (7) |  |  | 5.8% (42) | | 245 |  |
| Kunar | Chawkay | 100% (257) |  |  |  |  |  | | 273 |  |
|  | Nurgal | 100% (355) |  |  |  |  |  | | 145 |  |
|  | Assadabad | 97.5% (465) | 2.5% (12) |  |  |  |  | | 131 |  |
| Ghazni | Ghazni | 90.3% (242) |  |  |  | 9.7% (26) |  | | 294 |  |
|  | Khwaja Umary | 88.6% (156) |  |  |  | 11.4% (20) |  | | 234 |  |
|  | Qarabagh | 96.5% (305) |  |  |  | 3.5% (11) |  | | 123 |  |
| Badakhshan | Keshim |  |  |  | 1.3% (6) | 98.7% (472) |  | | 207 |  |
|  | Baharak |  |  |  |  | 100% (310) |  | | 97 |  |
|  | Fayzabad |  |  |  |  | 100% (209) |  | | 128 |  |
|  | **Total:** | **4022** | **1359** | **7** | **6** | **1048** | **54** | | **2880** |  |
