# Supplementary material for: Status of insecticide resistance in high-risk malaria provinces in Afghanistan
Source: Malar J. 2016 Feb 18;15:98. doi: 10.1186/s12936-016-1149-1 (PMC4758152; doi:10.1186/s12936-016-1149-1)
Supplement: Supplementary file 2 — 10.1186/s12936-016-1149-1 50 % and 90 % knock down time in minutes (KDT50 and KDT90 respectively) for DDT, permethrin and deltamethrin. [file 12936_2016_1149_MOESM2_ESM.docx]

Table S2: 50% and 90**%** knock down time in minutes (KDT_50_ and KDT_90_ respectively) for DDT, permethrin and deltamethrin

|  |  | DDT (4%) | |
| --- | --- | --- | --- |
| Province | Dominant species | KDT_50_ (CI 95%) | KDT_90_ (CI 95%) |
| Kunar | *An. stephensi* | 109.6 (4.507–4.887) | 272.5 (5.191–6.024) |
| Badakhshan | *An. superpictus* | 23.2 (3.090–3.199) | 37.5 (3.548–3.702) |
| Nangarhar | *An. stephensi* | 126.4 (4.538–5.141) | 470.1 (5.500–6.806) |
| Laghman | *An. culicifacies* | 147.0 (4.677–5.305) | 505.6 (5.567-6.884) |
| Ghazni | *An. stephensi* | 230.0 (4.863–6.013) | 1134.9 (5.891-8.178) |

|  |  | Permethrin (0.75%) | |  |
| --- | --- | --- | --- | --- |
| Province | Dominant species | KDT_50_ (CI 95%) | KDT_90_ (CI 95%) | |
| Kunar | *An. stephensi* | 30.9 (3.362–3.502) | 75.1 (4.201–4.044) | |
| Badakhshan | *An. superpictus* | 7.0 (1.723–2.178) | 18.6 (2.790–3.051) | |
| Nangarhar | *An. stephensi* | 27.6 (3.215–3.424) | 113.2 (4.509–4.949) | |
| Laghman | *An. culicifacies* | 20.4 (2.935–3.093) | 45.9 (3.715-3.939) | |
| Ghazni | *An. stephensi* | 10.3 (2.208-2.453) | 24.0 (3.064–3.295) | |

|  |  | Deltamethrin (0.05%) | |  |
| --- | --- | --- | --- | --- |
| Province | Dominant species | KDT_50_ (CI 95%) | KDT_90_ (CI 95%) | |
| Kunar | *An. stephensi* | 37.3 (3.534–3.707) | 122.2 (4.611–5.001) | |
| Badakhshan | *An. superpictus* | 14.1 (2.575–2.724) | 27.4 (3.212–3.410) | |
| Nangarhar | *An. stephensi* | 43.1 (3.660–3.865) | 164.3 (4.832–5.372) | |
| Laghman | *An. culicifacies* | 32.9 (3.414–3.575) | 97.4 (4.404–4.753) | |
| Ghazni | *An. stephensi* | 17.5 (2.787-2.931) | 34.5 (3.441–3.643) | |
